# Roads Less Traveled: Sexual Dimorphism and Mast Cell Contributions to Migraine Pathology

**DOI:** 10.3389/fimmu.2016.00140

**Published:** 2016-04-19

**Authors:** Andrea I. Loewendorf, Anna Matynia, Hakob Saribekyan, Noah Gross, Marie Csete, Mike Harrington

**Affiliations:** ^1^Huntington Medical Research Institutes, Pasadena, CA, USA; ^2^Department of Ophthalmology, Jules Stein Eye Institute, David Geffen School of Medicine, University of California, Los Angeles, Los Angeles, CA, USA; ^3^Brain Research Institute, David Geffen School of Medicine, University of California, Los Angeles, Los Angeles, CA, USA

**Keywords:** angiotensin, asthma, blood–brain barrier, estrogen, mast cells, progesterone, sodium–potassium ATPase, testosterone

## Abstract

Migraine is a common, little understood, and debilitating disease. It is much more prominent in women than in men (~2/3 are women) but the reasons for female preponderance are not clear. Migraineurs frequently experience severe comorbidities, such as allergies, depression, irritable bowel syndrome, and others; many of the comorbidities are more common in females. Current treatments for migraine are not gender specific, and rarely are migraine and its comorbidities considered and treated by the same specialist. Thus, migraine treatments represent a huge unmet medical need, which will only be addressed with greater understanding of its underlying pathophysiology. We discuss the current knowledge about sex differences in migraine and its comorbidities, and focus on the potential role of mast cells (MCs) in both. Sex-based differences in pain recognition and drug responses, fluid balance, and the blood–brain barrier are recognized but their impact on migraine is not well studied. Furthermore, MCs are well recognized for their prominent role in allergies but much less is known about their contributions to pain pathways in general and migraine specifically. MC-neuron bidirectional communication uniquely positions these cells as potential initiators and/or perpetuators of pain. MCs can secrete nociceptor sensitizing and activating agents, such as serotonin, prostaglandins, histamine, and proteolytic enzymes that can also activate the pain-mediating transient receptor potential vanilloid channels. MCs express receptors for both estrogen and progesterone that induce degranulation upon binding. Furthermore, environmental estrogens, such as Bisphenol A, activate MCs in preclinical models but their impact on pain pathways or migraine is understudied. We hope that this discussion will encourage scientists and physicians alike to bridge the knowledge gaps linking sex, MCs, and migraine to develop better, more comprehensive treatments for migraine patients.

## Introduction

Migraine is the most common neurological disorder, affecting 18% of females and 6% of males, with prevalence peaking at age 30–40 years. Since migraine triggers include stress, alcohol, menstrual cycling, missing meals, or sleep, it is not surprising that migraine prevalence peaks when other significant personal, family, professional, and financial responsibilities are also pressing. The female preponderance of migraine suggests that factors increasing female vulnerability and/or protecting males deserve greater consideration as contributors to migraine pathology.

The incapacitating features of migraine include episodic severe headache, accompanied by pain or severe discomfort in response to normal light, sounds, smells, touch, and often associated with nausea, vomiting, or vertigo. Symptoms are typically worse on movement and may last from 4 to 72 h, though a substantial number of sufferers (three million in the US) develop chronic daily headache (CDH). Only about 25% of migraineurs have a warning pre-headache aura, usually a short-lived, migrating visual hallucination. Common migraine comorbidities affect multiple organ systems in addition to the CNS (1). These include Raynaud’s phenomenon, hypertension, interstitial cystitis/bladder pain syndrome (IC/BPS), allergy and asthma, irritable bowel syndrome (IBS), osteo- and rheumatoid arthritis, anxiety, tremor, and depression (2–7). The molecular underpinnings common to and connecting these disorders are not known, but may include shared genetic risk factors (1, 8), regulation of brain cations (9, 10), or common receptor signaling events that activate pain (11), inflammation (12), or oxidative (13) pathways.

Treatment of migraine is multimodal, including lifestyle modifications, relaxation, yoga, physical therapy, massage, acupuncture, biofeedback, and cognitive-behavioral therapy, as well as prescription medications and over-the-counter supplements. Medications are directed at prophylaxis or rescue (14–17). A recent review describes rescue and prevention of menstrual migraine (18).

## Quantification of Pain

One barrier to effectively dealing with migraine, common to all chronic pain states, is quantifying the severity of “real” pain. Subjective metrics are difficult to translate across studies and objective measures fail to capture the true significance of pain. The best clinical practices require intensive patient–doctor dialog and individual patient education, also not easily translatable across studies. Useful clinical tools include FACES (developed for children), numeric (0–10), visual analog, and verbal pain scales. Descriptive terms (hot/cold, dull/sharp, and superficial/deep) can help classify pain (somatic, visceral, or neuropathic), and locations provide sensory discrimination often useful for diagnosis and treatment. There is a real unmet need for objective measures of pain. For example, electromyography (EMG) is used to measure ocular photic discomfort and facial grimace scores (an observation-based version of the FACES pain scale) (19, 20) [Kardon and Poolman, University of Iowa, VA Center of Excellence Iowa City, personal communication]. Brain mapping can identify brain regions activated in specific pain conditions, including migraine (21–24) and photophobia from corneal damage (25), but resolution is poor and not standardized for clinical use (26). The posterior insular-opercular, prefrontal cortex, and anterior cingulate cortex were identified as regions of interest for migraine in a meta-analysis of 22 migraine patients and controls using voxel-based morphometry. Notably, more women than men showed decreased gray matter in the dorsolateral prefrontal cortex (27). Ideal objective pain measures must be validated against clinical pain scales and must also reflect affective and motivational aspects of pain.

## The Pathophysiology of Migraine

**Figure 1:**
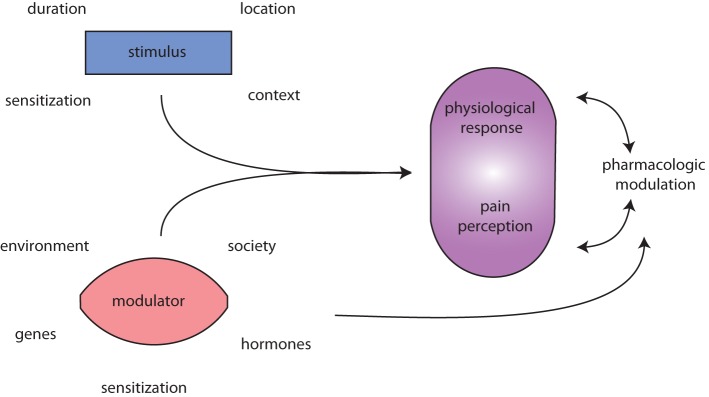
***Migraine is messy!* Migraine (and pain) triggers are numerous and highly variable**. The initiating stimulus depends on context (environmental or learned), location (eye strain, neck strain, and GI), type (chemical and mechanical), duration (short, long, or repeated exposure), and prior sensitization (extended drug use, allergies, autoimmune reactions, etc.). Modulators of stimuli, such as genetic predisposition, environmental factors, societal influences, and sensitizations, such as xenoestrogens, and endogenous sex hormones alter physiological responses to migraine and pain. Both the stimuli and modulators input to evoke both a physiological response (nociception) and interpretation of that response, pain perception. Pharmacologic treatment of (migraine) pain can modulate either or both the physiological response and pain perception. Additionally, pharmacological agents and lifestyle changes are also subject to the same modulators as the triggers.

In spite of the commonness of migraine, its burden on society, and WHO recognition of migraine in the world’s top 20 most disabling conditions (26), its pathophysiology is incompletely understood (Figure 1) (28). We do not know if a common pathway, component, or event is disrupted in migraineurs generally vs. in non-migraineurs, or whether migraine is really several diseases. Candidate mechanisms include cortical spreading depression (CSD) (29–31); dysregulation of neuropeptides (32); sterile meningeal neuroinflammation (33, 34) with triggering of dural mast cells (MCs) (35); altered central excitatory/inhibitory homeostasis (glutamate/gamma-aminobutyric acid) (36, 37); cortical neuromodulation (serotoninergic, noradrenergic, cholinergic, or dopaminergic) (37–40); channelopathy (41); or disturbed sodium homeostasis (42). Sterile meningeal neuroinflammation activates trigeminal primary afferents innervating the meningeal vasculature, providing a direct link to nociceptive circuits. Meningeal MCs are implicated in this mechanism, and dural MCs are directly activated in an animal model of migraine (35).

These candidate mechanisms also likely interact. For example, CSD is a slow, self-propagating transient wave of depolarization that suppresses activity in the cortex and is thought to underlie aura. CSD also increases meningeal blood flow and causes release of calcitonin gene-related peptide (CGRP), which may activate trigeminal nociception *via* the trigeminovascular system. CGRP, the main trigeminal pain mediator, is elevated in jugular blood during migraine (43). Antagonism of CGRP receptor (with olcegepant) and humanized antibodies against CGRP or its receptor are promising candidate migraine treatments.

### Central Sensitization in Migraine Pathology

Central sensitization (CS) represents enhanced signaling through nociceptive pathways (caused by increases in membrane excitability and synaptic efficacy as well as reduced inhibition). CS also implies loss of the normal remarkable plasticity of the somatosensory nervous system in response to activity, inflammation, and neural injury (44). CS presents clinically as allodynia (45, 46), can persist long after an insult (47), and can be visualized by functional magnetic resonance imaging (fMRI) (48, 49).

Not surprisingly, CS is also accompanied by changes in neurotransmitters. For example, serotonin and endocannabinoids are implicated in both depression and migraine (50, 51). Decreased urinary melatonin levels are reportedly associated with chronic migraine, depression, anxiety, and fatigue (52). Migraine and many of its comorbidities share alterations in serotonin (53), noradrenaline (54–56), estrogen (57), cannabinoids (58, 59), phosphocholine-specific phospholipase C (60), and glutamate (61, 62). Medications that modulate the G-protein-coupled receptors (GPCRs) for these ligands can sometimes alleviate symptoms of both migraine and comorbidities (63, 64).

### Channelopathy and Sodium Homeostasis Disturbance in Migraine

Channelopathies that alter ion homeostasis are implicated in familial hemiplegic migraine (FHM) (but not in migraine generally) as autosomal dominant mutations affecting calcium and sodium ion channels and the Na,K-ATPase transporter (65).

Ion fluctuation in CNS is exaggerated during migraine, with increased sodium concentration in the rat brain interstitial fluid during CSD (66), in cerebrospinal fluid (CSF) but not blood during migraine in humans (10), and in the brain and eyes after nitroglycerine (NTG)-triggered CS (42). These various models suggest that increased extracellular sodium, well known in CSD (66), is important in migraine and CS.

The primary control over sodium homeostasis in the nervous system is Na,K-ATPase (67, 68) that catalyzes transport of Na and K across cell membranes. Na,K-ATPase dysregulation at the neuronal and axonal plasma membrane generates abnormal local extracellular [K^+^] and intracellular [Na^+^] resulting in abnormal resting membrane potentials, axonal conduction properties (69, 70), and neuronal excitability (71). A knock-in mouse model with the FHM type 2 mutation of one Na,K-ATPase isoform has a decreased induction threshold for CSD (72). These mice do not demonstrate sexual dimorphism with regard to CSD propagation.

Dietary sodium intake, however, differs by sex in rodents, with females drinking more 3% NaCl than males. This pattern is established neonatally and can be suppressed in adult rats by testosterone (73). Neonatally androgenized females display low male-like salt intake and neonatally gonadectomized males display female-like high sodium intake (74, 75).

## Pain and Sex

Fundamental differences in pain perception from person to person make objective pain measurement difficult, but it is generally accepted that males and females experience pain differently. A general impression is that women have lower pain thresholds but higher tolerance, they seek treatment and discuss pain more than men, take fewer medications, and have a higher level of daily functioning and adaptation to pain (73, 76), though this impression is not universally accepted. Importantly, differences in pain response are both biological and psychosocial, and clinical studies are not often designed to capture sex differences (76). Sex hormones are certainly involved, but other genes, for example, SRY on the Y chromosome also underlie differences in pain experience (77). Obviously, pain studies only performed in men will not necessarily translate to women, if pain is influenced by sex hormones. It is also difficult to determine if women take less opioids because of greater analgesic sensitivity or decreased tolerance of negative side effects? Considering that women predominate in chronic pain conditions [reviewed in Ref. (78)], female-focused studies should be emphasized more. Though appropriate study populations may naturally follow from patient enrollment, the theoretical and practical design of research should be sensitive to sex differences.

### All Pain Is Not Created Equally

The source or location of pain is important in sexual dimorphism of pain. In a prospective interventional study using needle EMG, women rated pain higher than men, although both reported only “moderate” pain. Different muscles were associated with different pain levels, although the authors did not report if reported pain was higher in all or some muscle groups for women (79). A study of thermal pain showed the same sensitivity thresholds for men and women, but greater tolerance in men (80). Meta-analyses of pain studies are limited by small numbers of subjects, but can identify relevant features of pain: for example, in a meta-analysis of pain and analgesic requirements after ophthalmic surgery, three of four studies showed that no difference in pain by sex but a single study, including more participants than the other three, found females experienced more pain (81). Sex-based differences are not surprising given widespread expression of estrogen, progesterone, and androgen receptors in the eye (82) and brain. Notably, several ocular diseases show sex hormone-dependent changes in frequency, including glaucomas, dry eye, and central vein occlusion, but ocular pain itself is understudied in this regard (82). A study of over 15,000 patients having spinal surgery for lumbar disk herniation found more women consumed analgesics than men, reported higher levels of leg pain, lower quality of life, and higher disability (83). A meta-analysis of chronic widespread pain also showed higher incidence in women, particularly in peri- or post-menopausal women (84). Unfortunately, then, the literature contains no clear message about sex and pain, likely because of design issues, inadequate study size, retrospective design, types of surgeries analyzed, and pre-existing conditions, and difficulty quantifying pain. Clearly, we have work to do in studying pain in as standardized, objective, and controlled a way as possible (85). Even the attractiveness of the examiner can influence the pain rating by subjects (86).

### Pain in Animal Studies

Useful metrics of pain for animal studies are indirect and require inference from observed actions, and cannot capture motivation behind behaviors. Behaviors that reflect pain include avoidance responses (tail withdrawal, foot removal from thermal or mechanical stimuli, blinking, and light aversion), or tending to the site of pain (licking, foot shaking after formalin injection, head grooming in migraine models, and rubbing eyes for dry eye).

Fortunately, animal studies are generally consistent with human studies. Lower thresholds for pain and increased opioid requirements for analgesic effect (behaviors) have been shown in females vs. males. Opioid efficacy also varies by genetic background in mice (87), which can reflect alterations in mu opioid receptor (μOR) binding and signaling, μOR SNPs (single nucleotide polymorphisms) or alternative molecular pathways (88). Sodium affects the stability of antagonist conformation of GPCRs, including opioid receptors (89). These observations point to studies that can lead to improved and appropriately targeted therapies.

## Estrogen and Testosterone Effects in Human Migraine Animal Models

One-third to one-half of female migraineurs report worse migraines peri-menstrually, and ovulation can be a trigger. Migraine tends to lessen during pregnancy and lactation, and after menopause strongly implicating female hormone fluctuations as triggers. These observations have led to the use of both rescue and prophylaxis with all migraine-approved medications around the time of menses. Hormone therapy to reduce estrogen fluctuations may help migraine suffering, but are used cautiously because of the small increased risk of stroke in patients with migraine and aura (90). Anecdotally, changes from one birth control pill formulation to another can worsen or improve migraine. Animal experiments also support a role for sex hormones in migraine pathogenesis: female mice were more easily centrally sensitized than males, oophorectomy rendered the sexes comparable, and estrogen replacement to oopherectomized animals partially restored pain over-sensitivity (91). In male mice, orchiectomy increased CS, which was partially reversed with testosterone replacement (92). The target cells for these sex hormone actions are not clear, but imaging (93) and animal biochemistry studies (94) suggest that estrogens generally have excitatory actions on the brain while progesterone is inhibitory. (And of course the balance between them will be important.) Estrogen and progesterone increase CSD in rat cortical slices (95), possibly through modulation of excitatory glutamate release (96). Estrogen receptors are expressed in a wide variety of CNS neurons and in astrocytes; estrogen plays important and complex roles in synaptic function and neuroplasticity (97). Immune cells mediate differential mechanical pain hypersensitivity in male vs. female mice (98). Olfactory exposure to male investigators, their shirts, or to androgens and similar chemicals volatilized on sterile gauze, influences analgesia (99), and further emphasizes sexually dimorphic responses relevant to migraine.

## Sex-Based Differences in Drug Responsiveness

A U.S.-wide survey showed that more women than men used over-the-counter and prescription medications (100). A national Italian survey found no difference in the use of NSAIDS or ergot derivatives but increased use of triptans in women (101) for migraine. In a Finnish population, women were prescribed more drugs of all categories to treat migraine (73). But females were at greater risk of migraine headache recurrence than men (102). Whether these drug use patterns represent differences in migraine severity, requests by patients, or perceptions of the attending clinician is unclear but the literature suggests that gender is a factor in how migraine is diagnosed and treated [reviewed in Ref. (78, 86)].

Migraine pain medications prescribed in emergency departments (ED) do not differ between men and women, with 35% treated with opioids and 1.5% treated with triptans (103). These data represent from 58,000 civilian and 9.9 million military records, but the data are not rich enough to parse out sex differences in the quality of pain or efficacy of treatment. Nonetheless, they highlight a real problem with ER treatment of migraine. Opioids may reduce migraine pain acutely, but should be avoided as they can cause episodic migraine to become chronic (104).

Opioid therapy is a notable example of sex-based differences in drug responses at many different levels. Imaging studies show differences in the response of females to μOR agonists in ligand internalization, receptor distribution, and hormonal influence. Though morphine pharmacokinetics are similar in men and women (105), PET scans show brain-region-specific differences in the magnitude of endogenous μOR activation: men have increased activation in anterior thalamus, ventral basal ganglia, and amygdala; women have reduced activation in the nucleus accumbens (106), and many studies show that females require more morphine for analgesia than males, regardless of the type or source of pain [reviewed in Ref. (107)].

### Structural and Molecular Basis of Opioid Sexual Dimorphism

The mechanisms by which opioids cause transition from episodic to chronic migraine are important to understand for getting to the heart of migraine pathology. These mechanisms include increases in brainstem and cortical hyperexcitability (104). Chronic opioid administration leads to downregulation of the glutamate transporter on astrocytes, increasing the residence time of the neurotransmitter in the synaptic cleft, effectively increasing the duration of receptor activation and potentiating signaling. In cultured microglia, morphine upregulates brain-derived neurotrophic factor (BDNF), which is known to act on the NMDA receptors and influences mood disorders (108). Estrogen may play a role in this process since it mediates alterations in glutamate signaling (and glutamate modulation of CSD), uncouples μOR from downstream signaling, and estrogen metabolites are TLR4 agonists (109).

Imaging in rodents using markers of neuronal activation to either morphine or inflammatory pain show more periaqueductal gray neurons are activated in females, but fewer activated neurons project to the rostral ventromedial medulla, a circuit important for endogenous and exogenous opioid analgesia (86, 107, 110). Like CSD, this is a sex-hormone-specific effect since males feminized at birth show reduced response to morphine (111).

The estrous cycle influences both morphine-induced analgesia (greatest potency when estradiol is low) and release of CGRP (107). Estrogen can uncouple μOR from its downstream GPCR kinases and induce internalization (112). Understanding the molecular underpinnings of estrogen effects on opioid responses is important, but despite all these data and the extensive experience with morphine, it is still not possible to make general sex-based clinical recommendations for morphine administration (86, 107) in non-migraine pain states.

A surprising interaction occurs between morphine and TLR4, a receptor that recognizes bacterial lipopolysaccharide (LPS) and is localized on CNS microglia. Microglia secrete various cytokines, including IL-1, TNFα, and IFNγ, to not only neutralize bacteria but also influence pain patterns, including allodynia and hyperalgesia (113). Minocycline is a broadly used antibiotic that reduces microglia activation, indicated by reduced cytokine release (e.g., IFNγ, TNFα, and IL-1β). Minocycline potentiates morphine analgesia (113, 114), decreases p38 MAPK activation in spinal microglia (115), suppresses morphine-induced respiratory depression (114), and attenuates a morphine-dependent increase in cyclooxygenase-1 expression in cultured microglia (114). Morphine activation of TLR4 is blocked by naloxone, the μOR antagonist (116). Morphine-3-glucoronide (M3G) is a morphine metabolite that does not bind the μOR but opposes analgesic effects and enhances pain. The pain-enhancing effects of M3G can be blocked by minocycline, and IL-1 receptor antagonist and both isomers of naloxone, showing that μOR was not the direct target of minocycline (116).

Other sexually dimorphic effects related to morphine–TLR4 interactions link sex and the immune system. Intrathecal injection of LPS generates pain responses only in male mice. Removal of male sex hormones by castration reduced pain levels to those of saline controls and females, but testosterone replacement therapy in males and oophorectomy in females restored “normal” pain responses (117). Furthermore, male mice with a loss of TLR4 function display reduced pain responses typical of females. Importantly, sexually dimorphic pain responses were specific to the inducing agent and site of delivery (117). Spared nerve injury, and intrathecal injection of LPS or CFA resulted in similar sexually dimorphic responses whereas intrathecal zymosan, intracerebroventricular (ICV), or intraplantar injections of LPS elicited equivalent pain responses in males and females (117).

## The Role of Mast Cells in Pain

Mast cells, a critical component of the innate immune system, are large phagocytic cells from the hematopoietic lineage. They circulate as immature cells, then mature after they settle in a tissue. In the developing brain, they localize along meningeal blood vessels (BVs) (118) and contain the vast majority of brain histamine (119). In adult life, MCs are capable of migrating across an intact blood–brain barrier (BBB) (120). In the periphery, MCs are located in various tissues, and relocate in response to inflammatory cues. They are physically associated with nerves in animals and man (121–123). In bladder, for example, ~75% of MCs are in proximity to nerve fibers facilitating nerve-immune cell communication (124). MCs are a critical component of migraine as well as migraine comorbidities (Figure 1). The initiating factors for CNS-immune system co-activation are not known, but their interactions appear to perpetuate disease (pain) in a feed-forward fashion.

### Mutual Activation of the Nervous System and Mast Cells

**Figure 2:**
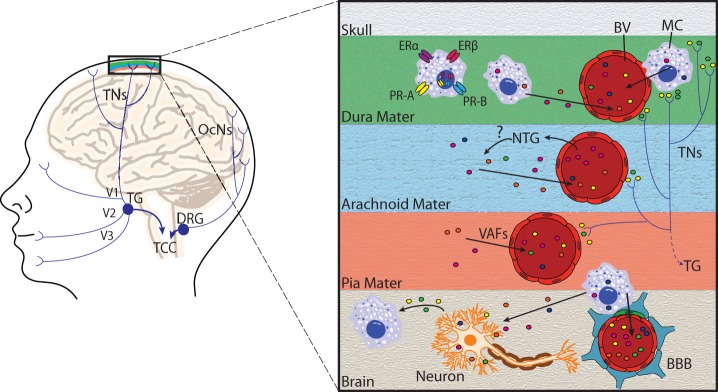
**Principal cephalic pain pathways and meningeal mast cell activation in migraine**. Left: the initiation of migraine headache follows activation of nociceptors innervating meningeal blood vessels. Pain information flows from these nociceptors *via* the trigeminal nerves (TNs) to the trigeminal ganglion (TG), which receives input from the meninges mainly *via* the ophthalmic branch of the trigeminal nerve (V1), and to a lesser extent from the maxillary (V2) and mandibular (V3) divisions. Pain information is then transmitted to the trigeminocervical complex (TCC), which comprises the C1 and C2 dorsal horns of the cervical spinal cord and the caudal division of the spinal trigeminal complex. The occipital cervical nerves (OcNs) sense posterior head and neck pain (common in migraineurs). These pain signals traverse the dorsal root ganglion (DRG) where they also terminate in the TCC. Right: an enlarged view highlighting mast cell activation within the meninges and brain. Activation of meningeal nociceptors leads to the release of vasoactive proinflammatory peptides, such as calcitonin gene-related peptide and substance P from terminal nerve endings (colored circles near terminals), resulting in meningeal BV vasodilatation, and local activation of dural mast cells (MC). Mast cell estrogen receptors ERα and ERβ, and progesterone receptors A (PR-A) and B (PR-B) are located at the plasma membrane or in the nucleus, and mediate mast cell responsiveness to these sex steroids. Following mast cell degranulation by either meningeal nociceptor activation, [or experimental nitroglycerine (NTG) injections], mast cells secrete vasoactive factors (VAF) and cytokines, such as nitric oxide, TNFα, vasoactive intestinal peptide, and histamine (depicted by colored circles) in meninges and brain. Mast cells can also react to neuronal stimuli, including substance P, CGRP, corticotropin-releasing hormone, and histamine. Mast cell degranulation can also lead to disruption of the brain–brain barrier (BBB), which is depicted by astrocytic end feet (blue) and pericytes (green) that directly appose brain capillaries.

**Figure 3:**
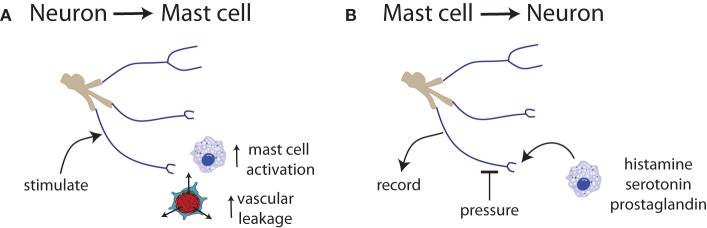
**Communication between neurons and mast cells is bidirectional**. **(A)** Trigeminal nerve stimulation results in mast cell degranulation and increased vascular leakage (131, 132). **(B)** Exposure of nerve endings to histamine, serotonin, or prostaglandin induces spontaneous activity and increased responsiveness to mechanical stimuli (133).

Upon activation, MCs secrete vasoactive mediators and cytokines, including nitric oxide (NO), TNFα, vasoactive intestinal peptide (VIP), and histamine (125–129) (Figures 2 and 3). In turn, MCs react to various neuronal stimuli, such as substance P (SP), CGRP, corticotropin-releasing hormone (CRH), histamine, many of which are also associated with migraine pathophysiology (119, 130).

The physical interaction and communication between nerves and MCs is mediated by adhesion molecules, such as cell adhesion molecule (CADM1) or N-cadherin (134–136). Communication between MCs and distant neurons occurs *via* transgranulation or release of exosomes (137) with granule-filled pseudopods cast off on the surface of the adjacent cell. Exosomes, secreted from multivesicular bodies and fusion with the plasma membrane, released by MCs can then target more distant cells with their protein and RNA cargos (138–140).

### Mast Cells and Pain Pathways

Mast cells interface in different locations outside of the CNS with nociceptors, predominantly with C-fibers. C-fibers are small, non-myelinated peripheral nerve fibers that detect noxious stimuli acid or chemical irritants (141). Once activated, C-fiber signaling is processed in the CNS to generate perceptions, such as pain, itch, urge to cough or sneeze, or subconscious activation of preganglionic autonomic neurons. For example, nociceptor activation in the gut can lead to secretion, diarrhea, and visceral pain (142–144). Additionally, local, autonomous afferent–efferent synapses independent of CNS control (“peripheral reflexes”) can transmit signals detected by a sensory nerve directly to nearby efferent enteric neurons, in gut, gallbladder, and airways (145). Neuropeptide-containing afferent C-fibers also directly regulate organ function *via* “axon reflexes” (146). “Axon reflexes” require the action potential of a peripheral nerve to travel until it reaches a bifurcation of the same nerve, then travel antidromically to endings of the same nerve. Once the action potential arrives, sensory neuropeptides, such as SP, neurokinin A, and CGRP, are released and can induce edema, vasodilation, smooth muscle contraction, and immune cell recruitment and activation. Thus, activation of a peripheral nerve can result in immune activation at the endings of the same nerve in a process termed “neurogenic inflammation” (146).

Mast cells are involved in pain in two ways: they secrete substances that directly activate or sensitize nociceptors (Figures 2 and 3). MCs release algogenic substances that activate nociceptors contributing to neuropathic pain (147), including trigeminocervical and lumbosacral tactile hypersensitivity (148). Sensitization of nociceptors can be mediated by MCs *via* histamine binding to nociceptors or nerve growth factor that binds the high-affinity nerve growth factor tropomyosin receptor kinase A (trkA) receptor (149). TrkA signaling is central to neuroprotection and neuroplasticity. Additionally, MCs secrete chemoattractants that recruit other immune cells to the site that can release pro-nociceptive factors (150).

### The Role of Mast Cells in Cerebral Pain

**Figure 4:**
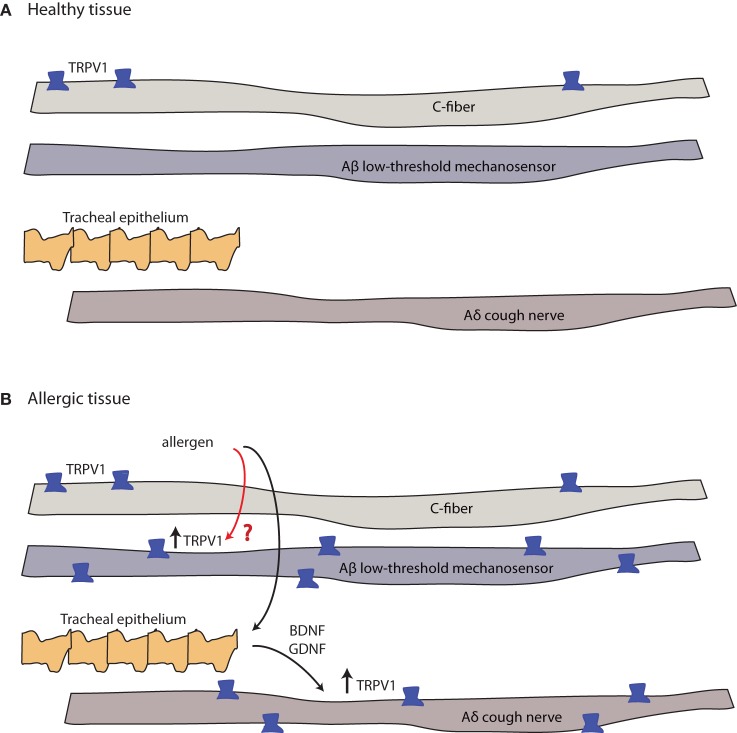
**Allergy-induced expression of pain receptors that may also occur in migraine and other pain conditions**. **(A)** C-fibers commonly express the ion channel protein transient receptor potential vanilloid 1 (TRPV1), while Aδ cough fibers and Aδ low-threshold mechanosensory fibers do not. Open channels allow cation (sodium and calcium) influx and action potential discharge (151, 152). **(B)** TRPV1-expression is stimulated by inflammatory eicosanoids, bradykinin, histamine, and others (4). Allergic sensitization with aerosolized allergen induces the expression of TRPV1 channels in both Aβ low-threshold mechanosensory fibers and Aδ cough fibers. In the latter, tracheal endothelium-derived brain-derived neurotrophic factor (BDNF) or glial-derived neurotrophic factor (GDNF) induce expression of TRPV1 mRNA (black arrow, known pathway), while the mechanism of the Aβ low-threshold mechanosensor fiber-capsaicin sensitization is unclear (red arrow, unknown pathway).

Several lines of evidence indicate mast cell involvement in cerebral pain (Figure 2). In electrophysiological studies, MC-derived serotonin, prostaglandin I_2_, and to a lesser extent, histamine were identified as sensitizing agents of meningeal nociceptors (Figure 4) (133). Interestingly, the usually inflammatory eicosanoid PGD2 and leukotriene C4 did not sensitize meningeal nociceptors (133). Nerve stimulation of rat trigeminal nerve (TN) resulted in increased vascular permeability, MC activation and degranulation in the orofacial area innervated by the trigeminal nucleus (131). Neonatal rats treated with capsaicin to deplete SP have the same MC activation, degranulation, and vascular leakage upon TN stimulation as untreated animals, suggesting that SP-mediated pain signals do not work *via* MCs (131). On the other hand, NO donor drugs cause enhanced CGRP release in trigeminal pathways, resulting in meningeal arterial vasodilatation and MC degranulation (32).

A subset of chemosensitive afferent nerves of the meninges express the protease-activated receptor-2 (PAR-2 receptor), and its activation also causes vasodilatation (Figure 4) (153). Proteolytic enzymes, such as tryptase released from MCs, can activate PAR-2 receptors inducing sensitization of the transient receptor potential vanilloid 1 (TRPV1) channel (153). TRPV1 is expressed in small nociceptive neurons, including in brain dura and trigeminal ganglia. Sumatriptan inhibits TRPV1 (154). Collectively, these observations suggest that MC activation and sensitization of chemosensitive meningeal and trigeminal ganglion (TG) C-fiber nociceptors may contribute to headaches (155).

Stress is a common trigger for migraines. Stress activation of brain MCs in rats is mediated, in part, by corticotropin-releasing factor (CRF) (130). Stress also selectively increases BBB permeability in rodents in brain regions rich in mast cells (156). Increased serum histamine levels in patients with migraines or cluster headaches further suggest MC degranulation during headache (157).

Primary MC disease (systemic mastocytosis or MC activation syndrome) is associated with symptoms of “brain fog”: loss of attention, focus, short-term memory, and ability to multitask (158, 159), underlining the connection between the innate immune system and the CNS.

### Mast Cells and Sex Hormones

Both estrogen and progesterone can activate MCs (160). Human and mouse brain MCs express estrogen receptors ERα and ERβ and/or the progesterone receptors A (PR-A) and B (PR-B) [reviewed in Ref. (160), Figure 2]. Steroid receptors are located at the plasma membrane or the nucleus (160). Upon binding hormone, nuclear steroid receptors form multiprotein complexes that determine whether the complex acts as a transcriptional repressor or enhancer (161). Steroid receptors located at the plasma membrane or on organelle membranes, by contrast, induce immediate signaling. Receptor location thereby determines the temporal relationship between hormone exposure and hormone-triggered effects. Nuclear receptor-mediated effects take minutes to hours, while plasma membrane and organelle-receptor mediated effects take seconds to minutes (160, 161).

Mast cell degranulation is a rapid response to estrogen or progesterone. Importantly, though androgen receptors are expressed in human MCs, testosterone does not mediate MC degranulation (162). In rats, MC densities in dura correlate with the availability of estrogen: males and ovariectomized females display the lowest density, while estrogen treatment of ovariectomized females increases dural MC density to that in intact females (163). MC numbers are modified by splenectomy, indicating that MCs migrate from spleen or that spleen promotes mobilization and migration of MCs (163). Though not well studied, it is also possible that MC phenotype is altered by the splenic environment as in other immune cells. Experimentally, MC degranulation is not impacted by either the estrus cycle of intact females or estrogen administration of oophorectomized females (163).

Other immune cells are also equipped with receptors for estrogen, progesterone, and other sex steroids but sex steroid effect on these cells is largely unstudied (164). So how other immune cells impact migraine is an open question, but their role in some migraine comorbidities is prominent. In allergies and asthma, allergen binding antibodies are a key component of the disease and all cells involved in the generation of antibodies (T cells, antigen-presenting cells, B cells, and regulatory T cells) express sex steroid receptors and can be modulated by these factors (164). Both the xenoestrogen bisphenol A (an environmental estrogen-like compound) and estradiol enhance differentiation of antigen-presenting cells that preferentially promote activation of T cells into the Th2 phenotype involved in antibody production (165, 166).

### Environmental Estrogens and Mast Cell Function

Mast cells have been extensively studied in the context of asthma, allergies, and anaphylaxis, largely with the help of well-defined rodent models. In those models, xenoestrogens promote MC degranulation and activation (167). Xenoestrogens are present in water and food in low concentration, concentrating up the foodchain and retaining bioactivity for long periods (167). In human, MC lines, xenoestrogens can induce MC degranulation, and their effects are additive with other xenoestrogens or estrogens (167, 168). Importantly, estrogen enhancement of degranulation was also observed in the setting of IgE-triggered MC degranulation characteristic of allergic reactions (167, 168). Xenoestrogen modulation of MC function in the context of diseases, such as migraine, has not been studied, but in a rat model, migraine behaviors were exacerbated by exposure to bisphenol A (169). Some researchers suspect that migraine incidence is increasing (170), and others have reported associations between air pollution and migraine (171) as well as urticaria reactions (172), raising questions about environmental pollutants generally in migraine triggering.

## Fluid Balance and Sexual Dimorphism in Migraine

Over-hydration or dehydration is common migraine triggers. Fluid homeostasis is influenced significantly by fluctuating gonadal steroids that change through the menstrual cycle or with hormone replacement therapy. Estrogen receptor expression is prominent in brain nuclei critical for maintaining fluid balance. The estrogen receptor ERβ is present in the vasopressin magnocellular neuroendocrine cells (MNCs) of the hypothalamus, while estrogen receptor ERα is present in the sensory circumventricular organs (CVO) (173). In hyponatremia, AVP release is strongly inhibited in the MNCs, while ERβ expression is increased (174, 175). ERβ expression is reduced in hyperosmolar states, in response to neuronal activation (175).

The renin–angiotensin–aldosterone system (RAS) plays a pivotal role in regulating blood pressure and fluid balance, targeting capillary endothelium and neurons throughout the brain (176) to regulate cerebral blood flow and stress responses (177). The angiotensin2 (AT2) receptor gene is regulated by estrogen and plays a critical role in blood pressure regulation in females (178). The RAS acts through the CVOs and the area postrema to activate pathways that elevate blood pressure, release vasopressin and aldosterone, and increase ingestion of water and sodium. The sensory CVOs, the subfornical organ, and organum vasculosum of the lamina terminalis (OVLT) lack a BBB and so are particularly sensitive to humoral signals, including plasma and CSF sodium, osmolarity, and AT2 levels (see below). Drugs that act on the RAS can help some migraineurs (179), but more extensive trials are needed to generalize these observations. High plasma ACE activity has been reported in the blood of migraineurs (180), suggesting that the RAS is disturbed. Nonetheless, ACE genotype polymorphisms have not been extensively studied for migraine therapeutic implications (181, 182). Activation of AT1 increases CGRP release from the dorsal root ganglia sensory neurons of spontaneously hypertensive rats (183), indicating another link between RAS modulation and migraine. Elevated plasma levels of CGRP found in migraineurs have led to development of CGRP antagonists and monoclonal antibody therapies with potential to rescue migraine (32).

## The Blood–Brain Barrier, Sex, and Migraine

The BBB is a highly selective unit that maintains brain homeostasis by limiting peripheral circulatory substances from entering the CNS (184). The functional neurovascular unit of the BBB is made of endothelial cells, pericytes, and astrocytes (185). Disruption in BBB function can lead to various neurological problems and has recently been implicated in pain disorders (186). The specialized tight and gap junctions between BBB endothelial cells prevent entry of toxins, and allow only small and lipophilic molecules into the CNS (185). The vascular theory of migraine, first articulated by Wolff (1948) implicates vasodilation of cranial arteries as the cause of the migraine pain (187). The role of the neurovascular unit has been heavily studied in relation to migraine but is still a source of debate (188–190).

The meninges are an interface between the systemic immune system and the CNS (Figure 2) with the relative immune privilege of the CNS maintained by the BBB. Traditionally, absence of a lymphatic system was also considered a key component of immune privilege. However, lymphatic systems in the immune privileged posterior eye, blind-ended were recently detected in humans (191). Additionally, a murine CNS lymphatic system was recently identified (192). This network is likely involved in CSF drainage, carries immune cells, and drains to the deep cervical lymph nodes. These recent advances in neuroimmunology are likely very relevant to migraine pathophysiology.

### The BBB and Migraine

Blood–brain barrier alteration in migraine is an area of intense study (193). In FHM type II, BBB disruption can be demonstrated using contrast-enhanced MRI (194). Other direct evidence of BBB dysfunction during migraine was observed in CSD rodent models. Increased cerebral cortex levels of metalloprotein 9 (MMP-9), a protease marker of BBB, were observed with associated edema and plasma protein leakage into brain (195). Increased plasma MMP-9 has also been implicated in migraine pathogenesis (196, 197). Women migraineurs have increased plasma MMP-9 concentrations during headache vs. interictal phases, and a particular MMP-9 haplotype (198). In order to compensate for a sudden substantial increase in CBF during migraine attacks, MMPs are thought to compensate by loosening TJs and expanding the BBB extracellular matrix (199), resulting in an inflammatory environment contributing to migraine (195).

Calcitonin gene-related peptide, a major player in migraine, causes dilation of the middle meningeal artery (MMA) in healthy volunteers, and sumatriptan reverses this dilation (22). Of note, pharmacokinetic parameters of triptans vary according to gender, with generally higher bioavailability in women and higher clearance rate in men (200). Gender-dependent dermal blood flow differences from capsaicin-induced release of CGRP (201) points to a specific mechanism underlying sexual dimorphism of migraine incidence.

## Migraine Comorbidities and Sex

Many of the extensive range of known migraine comorbidities and their overlapping genetic, molecular, and drug responses are reportedly more frequent in females (202), though not uniformly. One study showed that males tended to have more physical comorbid disorders, whereas females had more psychiatric comorbidities (8). Stress-related disorders are especially more common in females, including migraine, depression, and anxiety. Two groups of diseases with strong immunological components are also more prominent in women migraineurs: allergies and some autoimmune disorders. While environmental allergies are significantly higher in both men and women migraineurs compared to non-migraineurs, asthma is more frequent only in women migraineurs (202). Both osteoarthritis (driven by inflammation) and autoimmune rheumatoid arthritis are more prominent in women migraineurs. Similarly, different forms of autoimmune thyroid diseases are more prominent in women migraineurs than non-migraineurs, while male migraineurs are similar in this regard to male non-migraineurs.

In search of the basis for the sexual dimorphism of psychiatric disorders in women migraineurs, the locus coeruleus (LC)–norepinephrine (NE) neurons display more extensive dendritic arborization in female rats vs. male rats (203). Clinically, hormone fluctuations are clearly involved in peri-menstrual, menopausal, and postpartum depression. IC/BPS, IBS, and asthma are more common in females. Vestibular migraine is surprisingly common affecting about 1% of the population, and also has a female preponderance (204).

The neurovascular theory of migraine is in part supported by a positive correlation between Raynaud’s phenomenon and migraine (205). Systemic sclerosis is more common in females (206), as is hypothyroidism (207), and SLE, with potential sex-specific differences mediated by estrogen and its metabolites, decreased androgen levels, hyperprolactinemia, and gonadotrophic-release hormones (208). Prolactin, important in pregnancy and lactation, also affects angiogenesis, immune function, and osmoregulation in both sexes (209) and hyperprolactinemia has been linked to headache (210).

### Interstitial Cystitis/Bladder Pain Syndrome

Migraine is common in patients with IC/BPS. About 11% of women and 5% of men suffer from IC/BPS (211, 212) and, like migraine, it is more common in people with other pain conditions, including IBS and fibromyalgia, both more common in females (213, 214). Imaging has shown overlapping areas of involvement of brain regions of the salience, emotional response, and sensorimotor networks, and the prefrontal cortex in IC/BPS and other chronic visceral pain conditions (215, 216) IC is often diagnosed when urinary frequency, nocturia, and suprapubic pain are exacerbated by ovulation and under stress. Bladder MCs are activated in IC (217).

Bladder nerves include sensory afferents and autonomic efferents, and peptidergic, P2X-, and TRPV1 fibers from the lumbosacral dorsal root ganglion (DRG), along with adrenergic and cholinergic fibers. Numerous mechano- and nociceptive receptors have been described that respond to a variety of inputs, including EGF, SP, CGRP, CRF, acetylcholine, noradrenaline, adenosine, and inflammatory cells. Urothelial cells secrete many transmitters and mediators, including ATP (218), acetylcholine, prostaglandins, NO, and cytokines. Importantly, the bladder expresses both ERα and ERβ receptors and preclinical models show that E2 modulation of stress-activated kinase p38 MAPK varies during the menstrual cycle (219).

## Allergy and Asthma

Asthma and other allergic airway diseases are a prominent example of MC-driven diseases with higher propensity in females: similar to migraine, women are approximately three times more affected than men (220–222). Thirty to forty percent of women experience exacerbation of asthma symptoms peri-menstrually (223). Similar to cerebral pain sensitization patterns as a function of estrogen (91), oophorectomized animals have reduced airway inflammation (224) and tamoxifen (ER antagonist) treatment of intact females also reduced airway inflammation (224).

Mast cells activated by binding of allergen–antibody IgE complexes directly stimulate C-fibers that express receptors for many mediators present in the context of allergic disorders. This direct connection has been shown for airways, skin, gastrointestinal tract, and bladder (196, 197, 225, 226). Similarly, intestinal MC activation can sensitize spinal nerves such that a short-lived mechanical gut distension results in prolonged afferent nerve activation (227). This allergen activation of MCs can result in C-fibers hyperexcitability, lasting for several hours (228). Importantly, allergies, which trigger symptoms in the periphery, can also modulate CNS neurons, and contribute to CS (229). There is currently no evidence that CNS MCs are activated by peripheral allergen exposure and, thus, it is more likely that the strong peripheral nociceptor activity modulates CNS neurons *via* the release of peptides and transmitters at the central terminals of the afferent nerves. Thus, migraine and its prominent comorbidity, allergy, have a common denominator in MCs and triggering of one disease may exacerbate the other.

### Irritable Bowel Syndrome

GI inflammation is painful and leads to profound changes in CNS (230). Conversely migraine is more prevalent in patients with celiac or IBD than in the general population (231). GI inflammation invariably leads to disordered motility. With inflammation, neurons involved in peristalsis become hyperexcitable, and inhibitory neurotransmission is blunted (232). These changes can persist (in the spinal cord dorsal horn) even after GI inflammation has subsided. Abdominal migraine is distinct from inflammatory bowel diseases (or celiac disease or sickle cell disease that present in similar ways). Abdominal migraine pain is episodic and severe enough to prompt ER visits, most commonly in children. A case report suggests that pregnancy can interrupt the progression of abdominal migraine (233).

Mast cells contribute to IBS, a common comorbidity of migraine (234–236). Increases in intestinal neuron excitability, mesenteric sensory nerve activity, and visceral or somatic sensitivity possibly caused by intestinal mucosa serotonin, histamine, and MC tryptase contribute to IBS pathology (237) In the long term, the bowel nervous system responds by increasing expression of SP and TRPV1 and, therefore, bowel pain perception (132, 225, 238–240). MC infiltration of the colonic mucosa in IBS patients also correlates with the amount of released NGF, neuronal sprouting, and expression of the NGF receptor NTRK1 (241).

Other gastrointestinal motility disorders that share their comorbidity and a sexual proclivity for females with migraine include gastroparesis (242), cyclic vomiting syndrome (243), and infant colic (244). Colic is more common in offspring of mothers with migraine (245).

### Osteoarthritis and Rheumatoid Arthritis

Synovial inflammation is as an important feature of osteoarthritis and pain is its main symptom. Both peripheral and central neurological mechanisms are involved and, hence, osteoarthritis is considered a chronic nociceptive pain condition (246). In rheumatoid arthritis, joint pain is also the main symptom (247). Synovial inflammation in osteoarthritis presents with infiltration of macrophages, T cells, and MCs but the overall level of infiltration and cytokine production is lower than that in rheumatoid arthritis (248). The common denominator of osteoarthritis and migraine is nociceptive sensitization.

The pathogenesis of rheumatoid arthritis is not fully understood but involves both the innate and adaptive arms of the immune system eventually resulting in the breakdown of immune tolerance, autoantigen presentation, and both T and B cell activation (249). Interestingly, MMP-9 is a genetic marker of RA susceptibility and contributes to joint damage (250). As noted above, MMP-9 plays a role in migraine, and serum levels are generally elevated in migraineurs.

### Anxiety

Anxiety, including panic attacks, affects nearly one in five adults in the U.S. (251), but is even more frequent in migraineurs. Women are significantly more likely than men to develop an anxiety disorder (252). The National Comorbidity Survey (NCS, conducted from 1990 to 1992) found that lifetime prevalence rates for any anxiety disorder were 30.5% for women and 19.2% for men (253). Prevalence rates were also higher in women than men for each anxiety disorder examined (253, 254).

Anxiety is associated with an exaggerated sympathetic nervous system response, beginning with the hypothalamic–pituitary–adrenal (HPA) axis (255), with CRF secretion. The end products of this cascade are cortisol, epinephrine, and NE (256). Anxiety also results in glucocorticoid receptor resistance. Without appropriate cortisol regulation of the local cytokine response, there is greater susceptibility to compromised immune defenses (257).

### Depression Is a Common Migraine Comorbidity

Population-based and clinical studies have demonstrated the high prevalence of migraine and depression as comorbid disorders. Migraineurs are 2.2–4 times more likely to suffer from depression (258), and 28% of migraineurs experience major depressive disorder (MDD) (259), more common in migraineurs with aura (260). Rates of suicide attempt are also increased in migraineurs (261) and migraineurs with fibromyalgia. MDD patients with active migraine are particularly difficult to treat (262).

Moreover, migraine and depression are two to three times more common in women than men (254, 263). The relationship between migraine and depression appears to be bidirectional, suggesting a common neurobiology (264). Women with CDH are more likely to suffer from MDD than those with episodic headaches (265). Furthermore, episodic migraineurs with depression are at increased risk of developing chronic migraine (266). A few pathophysiological mechanisms linking the two disorders have been suggested: hormonal influences (58), serotonergic dysfunction, CS, and Na^+^,K^+^-ATPase dysregulation (9, 267, 268), all of which are controlled in part by estrogen or show gender-specific differences.

Women’s increased migraine attacks and mood disturbances have been linked with fluctuations in estrogen during menses, postpartum, and premenopausal periods (269). Anecdotally, estrogen augmentation can have potential positive therapeutic effects on depression (57). Physiological effects of estrogen span both disorders, including the modulation of neuropeptide Y, CRH, and the neurotransmitters serotonin, dopamine, and glutamate (57).

A serotonergic dysfunction associated with migraine has been linked with polymorphisms in the serotonin transporter 5-HT (270). An allele of 5-HT that slows serotonin synthesis is associated with increased susceptibility to depression and increased sensitivity to anxiety and stress (271). Chronically low serotonin is also implicated in CSD and heightened sensitivity of the trigeminovascular pathways of migraine (272). Depression and anxiety, associated with reduced serotonin are commonly treated with pharmaceuticals that increase central serotonin levels, some of which have also been used in treatment of migraine (273), thought SSRIs are not universally effective in migraine (15, 272, 274). Low-dose TCAs can also treat migraine, both rescue and prophylaxis in some patients (275) and other pain syndromes. Women tend to respond better to treatment with SSRIs due to hormone-dependent pharmacokinetics (276). Thus, anti-depressants, triptans, and anticonvulsants are often used in combination in treatment of both disorders (277, 278). Behavioral therapy also has a place in treating both migraine and depression (279) but despite these options, treatment of these two disorders is still challenging (280).

Allodynia, a clinical marker of CS, is correlated with increased risk of depression among migraineurs (281), and increased suicidal ideation (282). Pain perception is abnormal in patients with MDD, who display lowered pain threshold and tolerance (283).

As mentioned earlier, in some cases, migraine and depression progress into chronic states more refractory to treatment (262, 265, 266), suggesting a common CS syndrome, involving both emotional and sensory pathways (284, 285). Women have been shown to be more susceptible to stress and negative experiences, a well-known risk factor for depression and migraine (286). Chronic stress induces neuroinflammation (287). Activation or dysregulation of the HPA has also been implicated in migraine and depression (288, 289). Furthermore, estrogen receptors are abundant and localized in the hypothalamus. Animal studies have shown that females with high estrogen activity had a greater HPA axis response to stress compared to low-estrogen counterparts and males (290).

Inflammation is possibly the mechanistic link between depression and migraine, potentially due to proinflammatory cytokine alteration of tryptophan (TRP) metabolism, reducing 5-HT synthesis and activating the HPA axis (291). ER-β signaling increases 5-HT synthesis in murine brain (292). Clinically, depression symptomatology is linked to depletion of 5-HT and production of TRP metabolites by inflammatory cytokines (293). The role of CGRP in migraine, inflammation, and sexual dimorphism as noted above is also observed in patients with MDD (294).

Na^+^,K^+^-ATPase dysfunction in migraine has been discussed above. Reduced Na^+^,K^+^-ATPase activity contributes to both mania and depression (267, 295–297), likely through increased neuronal excitability and decreased neurotransmitter release (297). Mice lacking the Na^+^,K^+^-ATPase subunit FXDY2 have altered renal sodium handling and increased thermal instability (298). FXDY2 activity has also been proposed as a modulator of hypersensitivity to pain induced by inflammation (298).

## Conclusion

Common threads in migraine and comorbid conditions are sexual dimorphism, MC effectors, and neuronal hyperexcitability. We note these common features in hopes that immunologists, endocrinologists, and other domain experts in experimental and clinical neurosciences can inform each other. For example, the detailed understanding of MC dysregulation in asthma and allergy may help inform the pathogenesis of migraine. Awareness of the altered sodium homeostasis found in migraine may encourage studies of sodium homeostasis underlying some of the more common migraine morbidities that are often viewed within their clinical specialties as unrelated to migraine. Awareness of the prevalence of migraine, especially in women, should help inform the way histories are taken outside of neurology and headache clinics. Most importantly, understanding the common drivers of disease may help re-define the subtypes of migraine and its comorbidities: are MC-mediated migraines fundamentally different than those in which sodium dysregulation is a major driver? Current therapies are crude, non-specific, and must be highly individualized. Design of more rational therapies likely involves better phenotyping of migraine by gender-specific and other pathophysiological drivers of disease, and will certainly involve multidisciplinary input from psychiatry and pain specialists, allergists, and gastroenterologists, in dialog with neuroscientists.

## Author Contributions

All authors contributed to writing of the manuscript.

## Conflict of Interest Statement

The authors declare that the research was conducted in the absence of any commercial or financial relationships that could be construed as a potential conflict of interest.
